# Association of the rs1126616 and rs9138 Variants in the *SPP1* Gene among Mexican Patients with Systemic Lupus Erythematosus and Lupus Nephritis

**DOI:** 10.3390/ijms25021000

**Published:** 2024-01-13

**Authors:** Alicia Rivera-Cameras, Martha Patricia Gallegos-Arreola, María Cristina Morán-Moguel, Mario Salazar-Páramo, Miriam Fabiola Alcaraz-López, Gustavo Echeverría-González, Jorge Fernando Topete-Reyes, Sergio Adalberto Franco-Chávez, Ingrid Patricia Dávalos-Rodríguez

**Affiliations:** 1 División de Genética, Centro de Investigación Biomédica de Occidente, Instituto Mexicano del Seguro Social, Guadalajara 44340, Mexico; alicia.rivera3492@alumnos.udg.mx (A.R.-C.); marthapatriciagallegos08@gmail.com (M.P.G.-A.); 2 Doctorado en Genética Humana, Centro Universitario de Ciencias de la Salud, Universidad de Guadalajara, Guadalajara 44340, Mexico; 3 Departamento de Biología Molecular y Genómica, Centro Universitario de Ciencias de la Salud, Universidad de Guadalajara, Guadalajara 44340, Mexico; cristina.moran@academicos.udg.mx; 4 Departamento de Fisiología, Centro Universitario de Ciencias de la Salud, Universidad de Guadalajara, Guadalajara 44340, Mexico; msalazpa@hotmail.com; 5 Servicio de Reumatología, Hospital General Regional 46, Instituto Mexicano del Seguro Social, Guadalajara 44910, Mexico; fabiola_alcaraz@hotmail.com; 6 Servicio de Reumatología, Unidad Médica de Alta Especialidad, Hospital de Especialidades, Centro Médico Nacional de Occidente, Instituto Mexicano del Seguro Social, Guadalajara 44329, Mexico; gustavo.echeverria@imss.gob.mx; 7 Servicio de Nefrología, Hospital General Regional 46, Instituto Mexicano del Seguro Social, Guadalajara 44910, Mexico; fernando.topete@live.com.mx; 8 Departamento de Salud Pública, Centro Universitario de Ciencias de la Salud, Universidad de Guadalajara, Guadalajara 44340, Mexico; adalberto.franco@academicos.udg.mx

**Keywords:** Lupus nephritis, osteopontin, *SPP1* gene, systemic lupus erythematosus

## Abstract

Systemic lupus erythematosus (SLE) is a multisystem disease considered a prototype of the main autoimmune disease and presents serious complications, such as lupus nephritis (LN), which generates a significant impact on morbidity and mortality. The *SPP1* gene encodes the osteopontin (OPN) protein, which plays a crucial role in the regulation of inflammation and immunity. The variants rs1126616 and rs9138 of this gene have been associated with the inflammatory response. The study aims to analyze the association of the rs1126616 and rs9138 variants of the *SPP1* gene in SLE Mexican-Mestizo patients without LN (SLE-LN). In this cross-sectional study, a total of 171 genomic DNA samples from SLE patients were clinically confirmed, of which 111 were SLE without LN, 60 were SLE with LN, and 100 healthy individuals were included as reference group. The rs1126616 variant was genotyped using PCR-RFLPs, and the rs9138 variant was genotyped using qPCR TaqMan. The TT genotype, the recessive model [OR 2.76 (95% CI 1.31–5.82), *p* = 0.011], and the T allele [OR 2.0 (95% CI 1.26–3.16), *p* = 0.003] of the rs1126616 variant are risk factors for SLE with LN. By contrast, the rs9138 variant did not show statistically significant differences among SLE patients stratified by LN. In our study of SLE Mexican-Mestizo patients with and without NL, demographic and clinical characteristics do not differ from other SLE populations, and the TT genotype of the rs1126616 variant of the *SPP1* gene confers a risk factor for the presentation of LN. Otherwise, the rs9138 variant did not show association with NL.

## 1. Introduction

Systemic lupus erythematosus (SLE) is known to have a strong genetic component, so individuals with a family history of SLE are at a higher risk of developing the disease [1]. The prevalence of SLE in Mexico has varied over time and across regions. It is influenced by several factors, including genetics, environmental conditions, access to healthcare, and diagnostic criteria. Studies have estimated the prevalence of SLE in Mexico to be around 40 to 70 cases per 100,000 people [2]. The incidence of SLE in Mexico can also vary, and data on new cases are critical for understanding how the disease is spreading. However, precise incidence rates can be challenging to determine due to underdiagnosis, and not all cases are likely reported [3].

While genetics alone cannot predict who will develop SLE, specific genetic factors that contribute to susceptibility have been identified [4]. The *SPP1* gene encodes a glycoprotein called OPN, which is involved in a variety of physiological processes, including immune response, inflammation, and tissue repair. In the context of SLE, the *SPP1* gene and the OPN protein have been the subjects of extensive research for their roles in the disease’s development and progression [5]. OPN is a proinflammatory cytokine, and its overexpression is observed in the blood and affected tissues of SLE patients. High levels of OPN have been associated with the presence and severity of SLE; it contributes to immune dysregulation by promoting the activation of immune cells and the production of proinflammatory molecules and plays a role in the formation of autoantibodies, which are a hallmark of SLE [6]. Autoantibodies are produced by the immune system and target the body’s own proteins and DNA. OPN appears to influence the production of these autoantibodies and contributes to the chronic inflammation characteristic of SLE [7]. It is known that the immunogenetics of SLE is closely tied to abnormalities in immunological pathways. The aberrant activation of B-cells, T-cells, and dendritic cells, along with the dysregulation of cytokines, contributes to the autoimmune response [8]. SLE is characterized by the production of autoantibodies, which are antibodies that target the body’s own molecules [9]. Certain genetic factors influence the development of these autoantibodies, including defective B-cell tolerance and a genetic predisposition that can result in B-cells that fail to develop appropriate tolerance mechanisms, leading to the production of autoantibodies targeting nuclear components, such as DNA, RNA, and ribonucleoproteins. Otherwise, interferon signaling has been associated with genetic variants in genes such as *IRF5* and *STAT4*, which contributes to the overproduction of type I interferons, which stimulate immune cells to produce autoantibodies [9]. While genetic factors play a critical role in SLE susceptibility, environmental factors, such as UV light exposure, infections, and hormones, also interact with genetics to trigger the disease’s onset. Some individuals may carry SLE-susceptible genes but never develop the disease if they are not exposed to these environmental triggers [10].

Numerous studies have demonstrated that OPN encoded by the secreted phosphoprotein 1 (*SPP1*) gene is associated with the pathogenesis and progression of some autoimmune diseases [11,12,13,14,15,16]. Furthermore, recent investigations have provided new insights into the role of OPN in the pathogenesis of kidney diseases [17,18]. Previous studies indicated an association between rs1126616 and the predisposition of renal involvement, particularly in SLE. [19,20,21,22]. The rs9138 has been studied in SLE and is associated with a higher risk of SLE in males [16,23] and the clinical manifestation of photosensitivity [23].

Investigations revealed that rs1126616, as well as rs9138, is associated with higher OPN messenger RNA (mRNA) stability [24]. This synonymous variant can influence the stability of the mRNA that encodes the protein. Changes in mRNA can affect the amount of protein produced and its regulation [25]. This could influence how the gene’s expression is regulated and, therefore, the amount of OPN protein present in the body. Another study has suggested that the OPN protein can interact with other proteins and molecules in the body to carry out various functions. The variant rs9138 (+1239A/C) is in the *SPP1* gene [18] in the untranslated region at the 3′-UTR ends of the *SPP1*. The 3′-UTR is a crucial region in post-transcriptional gene expression regulation, as it contains elements that affect mRNA stability and translation. Genetic association studies have been conducted to determine whether this variant is associated with an increased risk of developing the disease or if it influences the severity of symptoms in SLE patients [26]. The relationship between rs9138 and SLE is not yet fully understood, though this variant may influence the expression or function of OPN. Although rs1126616 and rs9138 have been studied in different populations, no association studies have been reported with SLE in the Mexican population. With the above in mind, in this study, we analyze the association of the rs112616 and rs9138 variants, which are situated in the exon 8 and 3′UTR regions of the *SPP1* gene, respectively, in patients with SLE without LN and SLE with LN.

## 2. Results

### 2.1. Demographic and Clinical Characteristics

Demographic and clinical data concerning the SLE without LN patients, SLE with LN patients, and reference group are shown in Table 1. There were no differences in age and gender among SLE Mexican-Mestizo patients related to data that were similar in previous literature reported. The most frequent clinical criteria were mucocutaneous manifestations in 72.07% of SLE without LN patients and in 68.33% of SLE with LN patients. Arthritis was noted in 60.3% of SLE without LN patients and in 83.33% of SLE with LN patients, and hematological manifestations were observed in 55.85% of SLE without LN patients and in 81.66% of SLE with LN patients.

### 2.2. Analyses of the Variants

The analyses of the rs1126616 and rs9138 variants of the *SPP1* gene in the patients and reference group are shown in Table 2. The TT genotype and recessive model of the rs1126616 variant (OR 2.23, 95% CI 1.24–2.39, *p* = 0.001) were SLE risk factors. The rs9138 variant did not show statistically significant differences among the study groups. 

### 2.3. Comparative Analysis of the Allelic Frequency of the rs11267616 and rs9138 Variants of the SPP1 Gene in the Mexican-Mestizo Population (Reference Group with Different Populations)

**Figure 1:**
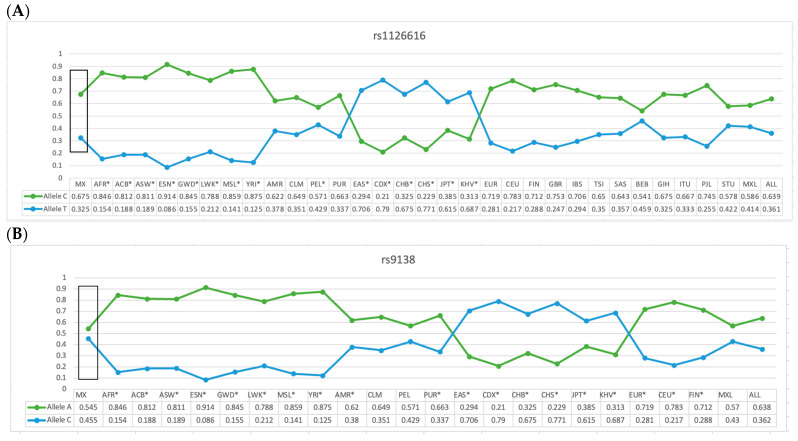
Allelic frequency comparison of the rs1126616 (**A**) and rs9138 (**B**) variants of the *SPP1* gene in the Mexican-Mestizo with reference group from other populations. MX = Mexican-Mestizo population from our study; ACB = African Caribbean in Barbados; ASW = African ancestry in the southwest US; ESN = Esan in Nigeria; GWD = Gambian in Western Division, The Gambia; MSL = Mende in Sierra Leone; YRI = Yoruba in Ibadan, Nigeria; CLM = Colombian in Medellin, Colombia; MXL = Mexican ancestry in Los Angeles, California; PEL = Peruvian in Lima, Peru; PUR = Puerto Rican in Puerto Rico; CDX = Chinese Dai in Xishuangbanna, China; CHB = Han Chinese in Beijing, China; CHS = Southern Han Chinese, China; JPT = Japanese in Tokyo, Japan; KHV = Kinh in Ho Chi Minh City, Vietnam; CEU = Utah resident with northern and western European ancestry; GBR = British in England and Scotland; BEB = Bengali in Bangladesh; ITU = Indian Telugu in the UK; STU = Sri Lankan Tamil in the UK. * Data with atheistic in Figure 1A,B represents the population that was different when compared with the data of the present study. Data are presented in Figure 1 and in the text; frequencies were taken from Ensambl, a database using the reference genome “CRCh38.p13.” The allele frequencies of different populations were taken from Ensambl. https://www.ensembl.org/Homo_sapiens/Variation/Population?db=core;r=4:87982201-87983201;v=rs1126616;vdb=variation;vf=90919299 (accessed on 29 October 2023) *. https://www.ensembl.org/Homo_sapiens/Variation/Explore?r=4:87982690-87983690;v=rs9138;vdb=variation;vf=90271607 (accessed on 29 October 2023) *.

The frequency of the C allele (rs1126616) and A allele (rs9138) of the *SPP1* gene variants in our reference group were statistically different when compared with groups from different populations around the world (*p* =< 0.05), as shown in Figure 1A,B.

### 2.4. Genotype Distribution of the rs1126616 and rs9138 Variants of the SPP1 Gene in SLE Patients, Stratified by LN

The distribution of both variants’ genotypes was studied in patients with SLE, stratified by the presence of the LN complication. The TT genotype and recessive model (OR 2.76, 95% CI 1.31–5.82, *p* = 0.011) and allele T (OR 2.0, 95% CI 1.26–3.16, *p* = 0.003) of the rs1126616 variant were SLE with LN risk factors. The rs9138 variant did not show statistically significant differences among the SLE patients stratified by LN. 

### 2.5. Comparative Analysis between Patients with SLE Stratified by LN with the Reference Group for the rs1126616 Variant of the SPP1 Gene

The comparative analysis between patients with SLE stratified by LN with the control group only showed differences with the TT genotype of the rs1126616 variant (OR 4.04, 95% CI 1.77–9.2, *p* = 0.001) as a risk factor.

The comparative analysis by age and gender among the study groups only showed differences with the TT genotype of the rs1126616 variant in male participants with SLE (OR 3.7, 95% CI 1.03–13.2, *p* = 0.047), compared to the control group. Likewise, in those patients with SLE with LN carrying the TT genotype (OR 3.6, 95% CI 1.1511.3, *p* = 0.042), in comparison with patients with SLE with LN and SLE without LN, regardless of gender.

### 2.6. Comparative Analysis between Patients with SLE with LN and SLE without LN for the rs1126616 and rs9138 Variants of the SPP1 Gene

The comparative analysis between patients SLE with LN and SLE without LN only showed differences with the TT genotype of the rs1126616 variant (OR 2.76, 95% CI 1.31–5.82, *p* = 0.011) as a risk factor, and the AC genotype of the rs9138 variant (OR 0.16, 95% CI 0.079–0.39, *p* = 0.0001) as a protective factor.

### 2.7. Haplotype Analyses of rs1126616 and rs9138 Variants of the SPP1 Gene in the Study’s Groups

**Figure 2:**
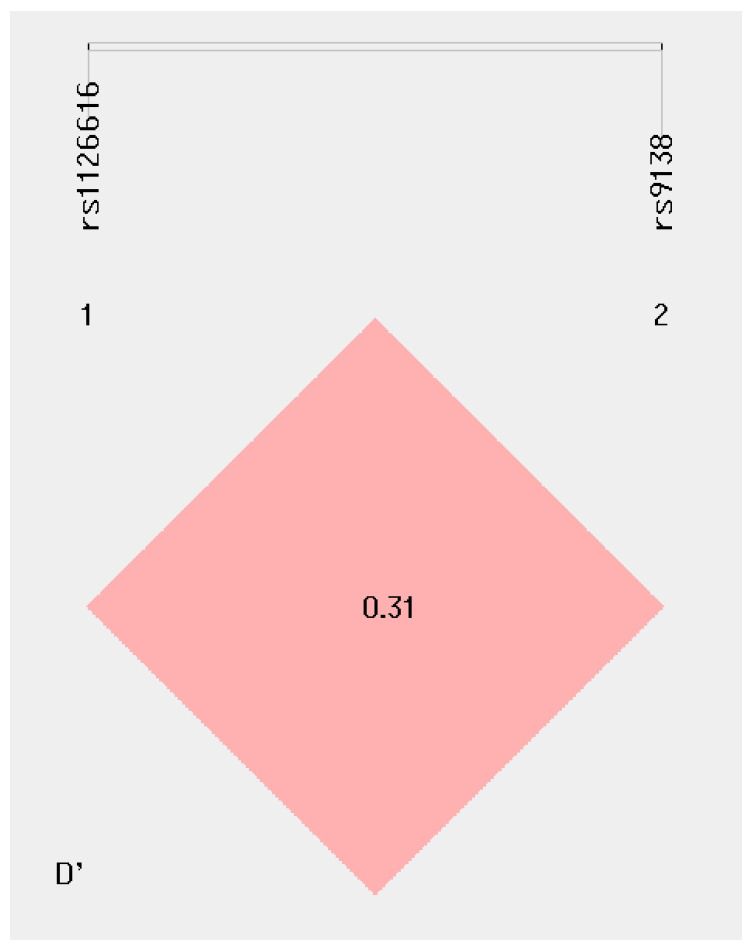
Haplotype frequency of rs1126616 and rs9138 variants of the *SPP1* gene.

Comparisons among the studied groups showed no statistically significant differences in terms of haplotype frequency. The linkage disequilibrium of the rs1126616 and rs9138 variants showed D’ 0.31 and r’ = 0.05; however, we observed that the frequency of the CA haplotype was higher in both patients and reference group (43%; 85/200), and in patients without stratification to LN (37%; 28/242); this was followed by the CC haplotype, which was noted in 25% of reference group and in 26% of patients with SLE, at 50/200 and 88/342, respectively (Table 3 and Figure 2).

The frequencies of the haplotype rs1126616 and rs9138 variants of the *SPP1* gene in patients stratified by SLE with LN and SLE without LN are depicted in Table 4, where statistically significant differences are observed in the TA haplotype in patients with LN (21%; 25/120) as a risk factor (OR 2.8, 95% CI 1.4–5.3, *p* = 0.002). By contrast, the frequency of the CC haplotype showed a statistically significant difference, with a protection factor (OR 0.4, 95% CI 0.25–0.79, *p* = 0.004) of 16% (20/120) in SLE with LN and 30% (68/222) in SLE without LN.

## 3. Discussion

Systemic lupus erythematosus (SLE) is a chronic and complex autoimmune disease that affects multiple organs and systems in the body. The etiology of SLE is multifactorial, with genetic and environmental factors playing an important role in its development. The heterogeneous clinical behavior of SLE is characterized by remissions and exacerbations, which present various complications, with LN being the most relevant complication due to the high risk of morbidity and mortality. The prevalence and incidence of SLE vary worldwide, with a higher incidence in women compared to men. Epidemiological data indicate that the prevalence of SLE is higher in African American, Hispanic, and Asian populations [27]. These numbers suggest a genetic component in susceptibility to SLE. LN, which is characterized by kidney inflammation, is a complication that can lead to severe kidney dysfunction and kidney failure; thus, it can have a significant impact on patients’ quality of life and survival. Even among Hispanic populations, which is how Latin Americans are usually grouped in clinical studies, there are differences in the presentation of the disease [28]. In our study, there were no differences with previous reports in the literature regarding the predisposition of the female gender in relation to the disease; the clinical presentations we observed revealed that the highest degree of involvement is the mucocutaneous in conjunction with arthritis; of the 171 patients, 60 presented with LN, accounting for 35.08% of the total population. Such a significant percentage denotes the recurrence of this complication and confirms that the incidence of the disease is in line with what is reported in the literature [29]. 

It has been estimated that nephropathy reaches rates of 39% among patients with SLE, and this condition is an important cause of morbidity and mortality [30]. In the present study, similarities were found with descriptions in the literature since the prevalence of the disease was significantly higher among females than in males (at a ratio of 9:1) and primarily affected patients of reproductive age [25]. 

Specific genetic variants within the *SPP1* gene, such as rs1126616 and rs9138, have been studied for their association with SLE susceptibility and severity. The *SPP1* gene is polymorphic and contains natural genetic variations that exist in the population. Among these variations are single nucleotide variants (SNV), such as rs1126616 and rs9138, which can influence gene function and expression [31]. These SNVs represent variations in the DNA sequence of the gene and can impact the expression and function of OPN. Research has suggested that certain genetic variants in *SPP1* may increase the risk of developing SLE. Additionally, these variants may be linked to a more severe disease course in those who already have SLE [31,32,33,34,35,36,37,38]. While the exact mechanisms by which these genetic variants influence SLE are still being elucidated, they play a role in modulating the immune response and inflammation in the context of this autoimmune disease [32]. Several studies in the literature have described similar results, with some highlighting the association of the rs1126616 variant with an increased risk of developing SLE with the T allele and others with susceptibility to the risk of developing major complications like LN [12,16,33,34,35,36,37,38].

We compared the allele frequencies of the variants rs1126616 and rs9138 in the control group and compared them with the allele frequencies of other populations, observing statistically significant differences in the AFR, ACB, ASW, GWD, LWK, MSL, YRI, PEL, EAS, CDX, CHB, CHS, JPT, KHV, and BEB populations regarding the rs112616 variant. We also observed significant differences when comparing the alleles of the rs9138 variant when comparing it with the allele frequency of our study’s participants when compared with other populations, resulting in differences with the AFR, ACB, ASW, ESN, GWD, LWK, MSL, YRI, AMR, PUR, EAS, CDX, CHB, CHS, JPT, KHV, EUR, CEU, and FIN populations related to both variants. This makes evident the genetic heterogeneity of *SPP1* and the importance of carrying out studies in each population.

In the present study, the TT genotypes and the T allele of the rs1126616 variant were associated with the risk of developing SLE without NL and SLE with LN (*p* < 0.05). The rs9138 variant did not show statistically significant differences among the study groups. Comparative analyses by age and sex among the study groups only showed differences with the TT genotype of the rs1126616 variant in males with SLE compared to the control group. Likewise, in those patients with SLE with LN carrying the TT genotype, compared to patients with SLE without LN, regardless of sex. The comparative analysis between SLE with NL and SLE without LN patients only showed differences with the TT genotype of the rs1126616 variant as a risk factor. We observed that the AC genotype of the rs9138 variant was associated with protective factors against the development of LN (*p* =< 0.05). This is the first study carried out in a Mexican-Mestizo population where this association of susceptibility to the risk of developing SLE and SLE with LN is evidenced. Previous studies carried out on SLE in other populations have focused on the rs1126616 and rs9138 variants.

A combined analysis of two ethnic groups showed the T allele of rs1126616 with a higher risk of susceptibility for SLE; likewise, a study of the Polish population [39] suggested the same association between the variant T allele and SLE susceptibility in rs1126616. Previous studies have reinforced the trend found in our western Mexican-Mestizo population, where the homozygous genotype had an association with SLE when compared against an apparently healthy population. We know that the biological meaning of a synonymous variant does not alter the amino acid sequence of the OPN protein [38]; however, this does not mean that the variant is biologically neutral. We propose theories about how this variant could affect in SLE, such as its impact on mRNA stability, despite not changing the protein itself. This synonymous variant may also influence the stability of the mRNA that encodes the protein. Changes in mRNA can affect the amount of protein produced and its regulation; otherwise, the rs1126616 variant could be in a regulatory region near the OPN gene [40]. This could influence how the gene’s expression is regulated and, therefore, the amount of OPN protein present in the body. Another study has shown that OPN protein can interact with other proteins and molecules in the body to carry out various functions. Although the amino acid sequence does not change, the variant could affect these interactions, which would impact the protein’s biological functions [41]. 

In addition to the above, a variant in the 3′UTR is not easy to identify; however, the results obtained in the present study are consistent with the observations made of the haplotype that include these variants and which have been related to the modifications in the protein [16]. The study by Forton et al. [21] was the first to propose rs1126616 in exon 7, significantly associating it with SLE. Later, it determined that two SNVs, rs7687316 and rs9138, contribute to SLE susceptibility [19]. The analyses of haplotypes (rs1126616, rs1126772, and rs9138) revealed that the association with SLE supports the hypothesis that the causal variant could affect the expression level of *SPP1* [41]. 

In recent years, there has been a greater appreciation of the importance of post-transcriptional regulation in eukaryotic organisms. The untranslated region at the 3′ end of a gene (3′UTR) is involved in the regulation of gene expression both at the pre-mRNA and the mature mRNA levels, playing a central role in the processing and polyadenylation of the mRNA, while the last cis elements in the 3′UTR are linked by transacting factors that modulate mRNA stability, nuclear and subcellular export, and translation efficiency [25,26,33,34,39]. Therefore, Martín et al. [25] concluded that polymorphisms in microRNA target sites within the 3′UTR can influence gene expression in complex phenotypes, such as lupus. For this reason, the *SPP1* variants rs1126616 and rs9138 make it a reasonable candidate gene for SLE association. The participation of the variants in the pathogenesis of SLE has been described in numerous articles wherein *SPP1* SNPs are associated with susceptibility and the overexpression of OPN [12,16,21,31,37,41]. In relation to the association of SLE with LN, which we observed as significant in our study, it stands out that [37] suggested that these SNVs in the *SPP1* gene are associated with a greater risk of developing LN and that the relationship between OPN and kidney damage goes directly to its biological function since, normally in humans, it is expressed in the Asa of Henle and in the proximal and distal convoluted tubule modulating angiotensin II-induced inflammation, oxidative stress, and fibrosis; thus, chronic kidney damage in SLE can be explained by the inflammation caused by the increase in OPN. This is why we consider OPN useful for a deeper understanding of the pathogenic processes of LN in SLE. We know that various causes can affect, such as medications and age, but in the present study, we observed a marked difference in expression independent of these external factors. The TT genotypes and the T allele of rs126616 are considered risk factors for the development of LN in patients with SLE.

Regarding the comparative analysis of the haplotypes (rs1126616 and rs9138), in the current study, we observed no significant differences when comparing the group of unstratified patients with the control group; however, based on a stratified analysis of the patients according to the presence or absence of LN, it was evident that the TA haplotype was shown as a risk factor when comparing patients with and without LN. In this regard, there are no studies in the literature where only the variants (rs1126616 and rs9138) are analyzed; however, the studies described herein demonstrate that the haplotype made up of rs1126616, rs1126772, and rs9138. The analysis of haplotypes rs1126616, rs1126772, and rs9138 revealed that the association with patients with kidney failure and the formation of calcium oxalate is likely associated with LN in the patients observed in the present study [37,41] T risk allele. SLE supports the hypothesis that the causal variant could affect the expression level of *SPP1*, as in recent years there has been greater.

Limitations of the present study are that the expression of the mRNA was not carried out, and there has been no analysis of the functional effect. Additionally, studies that include a greater number of patients are required. However, studies in populations of different ethnic origins are also relevant, such as the association analysis of *SPP1* variants in Mexican-Mestizo SLE patients which has not been previously reported [42].

## 4. Materials and Methods

A cross-sectional study was performed in Centro de Investigación Biomédica de Occidente, at the Instituto Mexicano del Seguro Social, in Guadalajara. All procedures performed in the study were done in accordance with the 1964 Declaration of Helsinki, and the participants provided their written consent. It was performed from 2021 to 2023. A total of 171 Mexican-Mestizo patients were included with a SLE diagnosis by EULAR/ACR 2019 and were stratified into two groups: 111 SLE without LN and 60 SLE with LN from the Rheumatology and Nephrology services of the Hospital General de Zona #46 and Rheumatology service of Centro Médico Nacional de Occidente, as well a reference group of 100 healthy population”. All subjects included in this study were considered Mexico-Mestizo and at least back to the third generation.

### Variant Analyses

The PCR amplification of the rs1126616 variant was performed using the following primers: F 5′ CCGTGGGAAGGACAGTTATG 3′ and R 5′ TTTAATTGACCTCAGAAGATGCAC 3′, as previously described [11]. These were performed in a total of 15 μL containing 0.2 mM dNTPs (Invitrogen, Carlsbad, CA, USA), 5 pmol of primers, 2.5 mM MgCl2, 2.5 U of Taq polymerase (Invitrogen, Carlsbad, CA, USA), and 50 ng of genomic DNA. The annealing temperature was 60 °C. The PCR product was digested with AluI restriction enzyme. In the previous electrophoretic procedure, amplified products were separated on 8% polyacrylamide gels (19:1), followed by silver staining. The C allele had an AluI cleavage site and was digested into 147 and 105 base pair (bp) fragments, while for the T allele, the 105 bp fragment was cleaved into 61 and 44 bp fragments. The rs9138 variant was identified by real-time PCR using the TaqMan probes 5′ TCTCATGAATAGAAATTTATGTAGA[A/C]GCAAACAAAATACTTTTACCCACTT 3′ (c___8826997_10), as designed and validated by Applied Biosystems (Thermo Fisher Scientific, Waltham, MA, USA). The reaction included a volume of 5 μL (~20 ng) genomic DNA, 6.25 microliters of TaqMan universal buffer, 0.32 microliters of VIC and FAM TaqMan-labeled probes, and 3.43 microliters of water per sample. They were all placed in 96-well plates in a light-covered system and were read using a C1000 touch Thermal Cycler, CFX96 Real-Time PCR System (BIO-RAD, Berkeley, CA, USA). As an internal control, 10% of the reactions were analyzed twice to observe concordance among all analyzed samples. The amplification conditions were as follows: 40 cycles at 95 °C for 10 min, then at 92 °C for 10 s, and at 60 °C for 1 min.

Allele frequencies were obtained by direct counting. A goodness-of-fit Chi-square test assessed the Hardy–Weinberg equilibrium to compare the observed genotype frequencies with the expected frequencies among control subjects. Odds ratios (OR) and 95% confidence intervals (CI) were calculated. A two-tailed *p* < 0.05 was considered statistically significant. The association analysis of the OR and the binary logistic regression analyses among the studied groups were performed using PASW Statistic Base 24, software, 2021 (Chicago, IL, USA). The SHEsis Online Version program analyzed pair-wise linkage disequilibrium (D′) and haplotype frequency.

## 5. Conclusions

The genetics of SLE are complex, and the *SPP1* gene and its product, OPN, are key players in the pathogenesis of the disease. OPN’s proinflammatory properties and their influence on autoantibody production make it a critical component of SLE pathology. Genetic variants in *SPP1*, such as rs1126616 and rs9138, have been associated with SLE susceptibility and disease complication. In our study of SLE Mexican-Mestizo patients with and without NL, demographic and clinical characteristics do not differ from other SLE populations, and the TT genotype of the rs1126616 variant of the *SPP1* gene confers a risk factor for the presentation of LN. Otherwise, the rs9138 variant did not show an association with NL.

## Figures and Tables

**Table 1:** Demographic and clinical characteristics of SLE patients and reference group.

	SLE without LN (*n* = 111)	SLE with LN (*n* = 60)	Reference Group (*n* = 100)
Age in years, mean (range)	42 (18–82)	42 (18–82)	41 (18–80)
Gender n, (%)	111 (100)	60 (100)	100 (100)
Female n (%)	98 (88.28)	48 (80)	50 (50%)
Male n (%)	13 (11.71)	12 (20)	50 (50%)
Clinical Criteria EULAR/ACR 2019 *			
Constitutional:			
Fever n (%)	1 (0.90)	3 (5)	-
Mucocutaneous n (%)	80 (72.07)	41 (68.33)	-
Arthritis n (%)	67 (60.3)	50 (83.33)	-
Neuropsychiatric n (%)	2 (1.80)	5 (8.3)	-
Serositis n (%)	30 (27.02)	20 (33.33)	-
Hematological n (%)	62 (55.85)	49 (81.66)	-
Asymptomatic n (%)	28 (25.22)	10 (16.66)	-

* https://www.ncbi.nlm.nih.gov/pmc/articles/PMC6827566/, accessed on 13 November 2023.

**Table 2:** Genotype and allelic distribution of the rs1126616 and rs9138 variants of the *SPP1* gene in SLE patients, SLE with NL, SLE without NL, and reference group.

Variants		SLE *		SLE with NL **		SLE without NL **		Reference Group *		OR *	95%(CI) *	*p* Value *
rs1126616	Genotype	(*n* = 171)	%	(*n* = 60)	%	(*n* = 111)	%	(*n* = 100)	%			
Model	CC	−82	48	−23	39	−59	53	−46	46			
	CT	−52	30	−17	28	−35	32	−43	43	1		
Model	TT	−37	22	−20	33	−17	15	−11	11	0.57	(0.34–0.96)	0.049
	CC	−82	48	−23	39	−59	53	−46	46	2.23	(1.08–4.61)	0.04
Dominant	CT+TT	−89	52	−37	61	−52	47	−54	54			
	TT	−37	22	−20	33	−17	15	−11	11	0.92	(0.56–1.57)	0.756
Recessive	CC+CT	−134	78	−40	67	−94	85	−89	89	2.23	(1.08–4.61)	0.04
	Alleles (2*n* = 342)			(2*n* = 120)		(2*n* = 222)		(2*n* = 200)				
	C	−216	0.6315	−63	0.525	−153	0.689	−135	0.675	0.82	(0.57–1.19)	0.353
	T	−126	0.3685	−57	0.475	−69	0.311	−65	0.325	1.21	(0–83–1.75)	0.353
rs9138								(*n* = 100)	%			
	AA	−38	22	−17	28	−28	25	−30	30	1		
	AC	−96	56	−34	57	−62	56	−49	49	1.33	(0.81–2.18)	0.312
	CC	−37	22	−9	15	−21	19	−21	21	1.03	(0.56–1.89)	1
Dominant	AA	−38	22	−38	22	−30	30	−30	30			
	AC+CC	−133	78	−133	78	−70	70	−70	70	1.5	(0.86–2.62)	0.154
Recessive	CC	−37	22	−37	22	−21	21	−21	21	1.03	(0.56–1.89)	1
	AC+CC	−134	78	−134	78	−79	79	−79	79			
	Alleles		(2*n* = 342)			(2*n* = 222)		(2*n* = 200)				
	A	−172	0.5029	−68	0.566	−118	0.5315	−109	0.545	0.84	(0.59–1.19)	0.391
	C	−170	0.4971	−52	0.434	−104	0.4685	−91	0.455	1.18	(0.83–1.67)	0.391

* Comparative analysis between SLE with the reference group. ** Comparative analysis between SLE with NL and SLE without NL, for the rs1126616 variant we observed significant differences comparing the TT genotype and TT recessive model in patients with NL and without NL with an OR 2.76 (95% CI 1.31–5.82) *p* 0.011, as well as for the T allele with an OR 2 (95% CI 1.26–3.16) *p* 0.003, however for rs9138 we did not observe a significant difference.

**Table 3:** Haplotype frequency of rs1126616 and rs9138 variants of the *SPP1* gene in SLE and Reference group.

Haplotype		SLE ^(2*n* = 342)^				Reference Group ^(2*n* = 200)^	
*rs1126616*	*rs9138*	*n*	%	*n*	%	OR 95% (CI)	*p*-Value
T	C	(82)	24	(41)	21	1.2 (0.80–1.8)	0.73
T	A	(44)	13	(24)	12	1.0 (0.63–1.8)	0.87
C	A	(128)	37	(85)	43	0.8 (0.56–1.1)	0.28
C	C	(88)	26	(50)	25	1.0 (0.69–1.5)	0.93

D’ (0.31) and r2 (0.05).

**Table 4:** Haplotype frequency of rs1126616 and rs9138 variants of *SPP1* gene in SLE without LN and SLE with LN.

Haplotype		SLE with LN ^(2*n* = 120)^				SLE without LN ^(2*n* = 222)^	
*rs1126616*	*rs9138*	*n*	%	*n*	%	OR 95% (CI)	*p*-Value
T	C	(32)	27	(50)	23	1.2 (0.74–2.0)	0.39
T	A	(25)	21	(19)	9	2.8 (1.4–5.3)	0.002
C	A	(43)	36	(85)	38	0.9 (0.56–1.4)	0.72
C	C	(20)	16	(68)	30	0.4 (0.25–0.79)	0.004

D’ (0.31) and r2 (0.05).

## Data Availability

All relevant data are included in the manuscript.
